# Hierarchical data structures for flowchart

**DOI:** 10.1038/s41598-023-31968-z

**Published:** 2023-04-09

**Authors:** Peng Zhang, Wenzhang Dou, Huaping Liu

**Affiliations:** 1 School of Software and Microelectronics, Peking University, Beijing, 100871 China; 2 School of Electrical Engineering and Computer Science, Oregon State University, Corvallis, OR 97331 USA

**Keywords:** Computer science, Information technology, Software

## Abstract

Flowcharts have broad applications in the fields of software development, engineering design, and scientific experimentation. Current flowchart data structure is mainly based on the adjacency list, cross-linked list, and adjacency matrix of the graph structure. Such design originated from the fact that any two nodes could have a connection relationship. But flowcharts have clear regularities, and their nodes have a certain inflow or outflow relationship. When graph structures such as an adjacency table or an adjacency matrix are used to store a flowchart, there is a large room for optimization in terms of traversal time and storage complexities, as well as usage convenience. In this paper we propose two hierarchical data structures for flowchart design. In the proposed structures, a flowchart is composed of levels, layers, and numbered nodes. The nodes between layers are connected according to a certain set of systematic design rules. Compared with the traditional graph data structures, the proposed schemes significantly reduce the storage space, improve the traversal efficiency, and resolve the problem of nesting between sub-charts. Experimental data based on flowchart examples used in this paper show that, compared with adjacency list, the hierarchical table data structure reduces the traversal time by 50% while their storage spaces are similar; compared with adjacency matrix, the hierarchical matrix data structure reduces the traversal time by nearly 70% and saves the storage space by about 50%. The proposed structures could have broad applications in flowchart-based software development, such as low-code engineering for smart industrial manufacturing.

## Introduction

Graph has a variety of use cases including hyperlink structure applications based on the world wide web, such as ranking of web search and application software based on web page and HTML protocols. Graphs require storage, traversing, processing and analysis, and some graphs contain millions or even billions of vertices and edges. Thus processing large-scale graphs based on the world wide web presents a significant technical challenge in terms of processing power and storage needs. To solve such a problem, there exist distributed graph algorithms such as MapReduce and accelerated solutions such as PageRank^1^. There are also research efforts that try to exploit the structures of the adjacency list and adjacency matrix to optimize the interactive processes between them^2^. Another direction is to exploit the data structure itself to optimize the design without changing the basic framework of the adjacency list and adjacency matrix^3^. These schemes could shorten the running time of the graph (especially web page graphs), save storage space, and improve graphs processing efficiency.

Arifuzzaman et al.^1^ analyzed the technical challenges that the adjacency list data structure is facing in representing large-scale graphs (e.g., those that are composed of billions of nodes and edges). Zhu et al.^2^ analyzed the graph data storage challenges from the perspectives of support transactions and edge scan efficiency. Lai et al.^3^ discussed the possibility of determining whether these exists one or more paths between two vertices of a graph via the adjacency matrix. They concluded that the path itself cannot be identified through the adjacency matrix, and proposed a method to determine the matrix path accurately. Kallaugher et al.^4^ studied the cycle-counting problem of the adjacency list data structure, and analyzed the space optimization issue of the adjacency list. Lin et al.^5^ described a distributed solution for graph data. Andersen et al.^6^ proposed an improved algorithm to compute approximate-PageRank-vector to improve the efficiency of graph data processing. Kang et al.^7^ proposed a scalable and general graph management and mining system, GBASE. It increases the query speed and thus reduces the query time without changing the graph data structure. Kiourtis et al.^8^ described an autoscaling big data analytics platform that users analytics through graph data modelling to support technical and nontechnical stakeholders.

Although there exist many solutions for large-scale graph data processing, these solutions still have obvious shortcomings in terms of efficiency because they require extensive hardware and software resources. For example, the work in^1^ tried to resolve this problem from the direction of distributed memory and parallel processing, and proposed a load balancing scheme and a parallel processing algorithm to improve space and time efficiencies. With the traditional adjacency list structure, the time and space complexities are ${{\mathcal {O}}}(\frac{e}{P}+n+P)$ and ${{\mathcal {O}}}(\frac{e}{P})$, respectively, where *n*, *e*, and *P* denote, respectively, the number of nodes, edges and processors.

There are also solutions designed for some particular types of graph, but they are essentially the traditional graph data structures such as the adjacency list and adjacency matrix.

Flowchart is a branch of graph and has many important applications. For example, experimental results reported in^9–11^ have shown that visual programming using flowchart could be very powerful, and in smart manufacturing, flowcharts are used to effectively describe production and manufacturing processes^12,13^. Siemens^13^ described Mendix, an application development platform for industrial manufacturing users. It includes app services that use flowcharts of binding code to make low-code or non-programming skills available. Taking automobile manufacturing as an example, the assembly process combines and connects a large number of parts according to the drawings and technical requirements. The sub-processes of the whole assembly process are not fixed, but vary based on specific needs. Each assembly process of automobile manufacturing can be abstracted as a node, and each assembly sub-process can be divided into several sub-nodes. Different assembly processes or different technological processes will generate different flowcharts. Thus one can associate nodes (assembly processes and technological processes) and edges (arrows that connecting two nodes) with computer program code blocks, and make them available to users in the form of web-version software. The software can run on the cloud, and is made accessible to users for smart manufacturing. Users can manipulate the flowchart required by the production processes via the web, and the computer in the factory can automatically complete the smart manufacturing process based on the codes associated with the flowchart. The integrated development environment (IDE) a software application that helps programmers develop software efficiently. Writing codes manually and repeatedly is inefficient and thus graphical symbols can be used instead of code blocks. Graphic symbols in a flowchart can be combined into several flowcharts, thereby drastically increasing the software development efficiency for many scenarios. Intelligent industrial manufacturing has led to the accelerated application of IDEs in production management software development. However, it has unique field-specific characteristics and requires a lot more software development talents than available in the field. Hence making industrial manufacturing software development easier and straightforward becomes critical. Flowchart is an efficient option to meet this need, since it can make very complex information and processes crystal clear.

However, creating a flowchart for complex processes could still be a very challenging task in terms of both time and storage complexities. The adjacency matrix path calculation method can also be applied to flowcharts, it has no advantages in terms of space and time complexities. Brunn et al.^14^ defined the adjacency matrix via the Collatz graph, and investigated various algorithms for generating the Collatz odd series. It showed that there is a room for optimization in using the adjacency matrix to store general flowcharts since their space and time efficiencies are very limited. Meesum et al.^15^ proposed graph-modification algorithms based on the adjacency matrix. Aiming for lower space and time complexities, they did not use structured methods to define graphs, but algebraic methods. This method works well for graphs with unstructured features, but it is not suitable for flowcharts that have structured flows and nested sub-processes. Aji et al.^16^ analyzed the characteristics of the anti-adjacency matrix (a transformed form of the adjacency matrix) and designed a characteristic polynomial to express the function of the number of vertices in a graph. They indirectly showed that the anti-adjacency matrix still has space and time efficiency problems when used to store flowcharts. Mehrpouya et al.^12^ introduced additive manufacturing that has provided significant freedom for creating complex components and highly customizable products. They also discussed the application of low-code development tool based on flowcharts in industrial manufacturing. Hooshyar et al.^17^ developed a flowchart-based programming environment (FPE) which provides the basic programming algorithms prior to surface structure using an automatic text-to-flowchart conversion approach.

For flowcharts with structured flows, how to efficiently represent them remains an unsolved problem. As artificial intelligence (AI) applications continue to develop at an accelerated pace, the uses of large-scale flowcharts are becoming explosive. This mandates new data structures with higher efficiencies to improve the decision-making and storage efficiency.

Existing research has shown that the logical data structure of the flowcharts still adopts the traditional adjacency list, adjacency matrix, cross-linked list and other conventional graph data structures. Due to the structured flow characteristics of flowcharts, applying these traditional graph data structures results in substantial redundancy in space and a high time complexity. Code platform being as concise as possible is vital for intelligent manufacturing, seamless coordination among autonomous vehicles, and dispatch of autonomous vehicles.

Graph visualization is a way for representing structural information as diagrams of abstract graphs and networks. It has important applications in networking, bioinformatics, software engineering, database and web design, machine learning, and in visual interfaces for other technical domains. A “concise-code platform” is a graph visualization software. It refers to the way experience and knowledge are presented to users via process and modularization of them. It improves the reuse efficiency of the back-end logics by simplifying the front-end, optimizes the iteration cycle, reduces cost, responds promptly to the outputs, improves the user experience, and makes application more convent. The idea of concise-code platform will promote broad applications of flowcharts with large scale of nodes. This will present a significant challenge toward a high processing efficiency of flowcharts.

The goal of this paper is to propose a new hierarchical data structure for large-scale flowcharts, leading to a much improved solution in terms of space (storage) and time efficiencies. For a single user (or small number of users) that requires small-scale flowcharts (e.g., those requiring 100 or so nodes), if the client/server (C/S) architecture is adopted, it is unnecessary to distribute the flowchart processing tasks to the client and servers, since it often results in insignificant time and space efficiency improvements. For a large number of simultaneous users, however, each user’s flowcharts will be processed in parallel on the server, resulting in huge amount of and extremely complex flowchart processing tasks. Challenges that large-scale graph data applications are facing are also discussed in^1^. Via some representative flowchart examples, the efficiency of flowcharts that use adjacency lists and adjacency matrices is shown to be poor. We propose an optimization method and a unique logical structure, fully exploiting the characteristics of the levels and layers of a flowchart.

The rest of this paper is organized as follows. Section “Data logical structure preliminaries” reviews existing data logical structures, covering basic concepts of adjacency matrix, adjacency list, cross-linked list. The proposed new hierarchical data structures for flowchart design are discussed in detail in Section “New data structures for flowchart design”. In Section “Analysis of the proposed data structures” we conduct an analysis of the proposed data structures, including traversal time and storage space comparisons with existing schemes. Concluding remarks are given in Section “Analysis of the proposed data structures”.

## Data logical structure preliminaries

Data logical structure can be classified into two categories: linear structure and nonlinear structure^18–20^. A linear structure has one and only one start node and one end node, and each node has at most one predecessor and one successor. A typical linear structure example is a linear table. A node in a nonlinear structure can have multiple predecessors and successors; if a node has at most one predecessor and multiple successors, then this structure is a tree structure; if there is no limit on the number of predecessors and successors of a node, that is, there may be an adjacency relationship between any two nodes, then this structure is called a graph.

Data’s logical structure describes the data from the logical relationship perspectives. It is independent of data storage and the hardware platform. It represents the user’s view and is a mathematical model for practical problems, which reflects the essence of how data are organized. Based on such structure, data can be flexibly organized for each specific problem, and all data elements can be optimized based on the required logical structure. The computer memory consists of a limited number of storage units, each of which has a unique address. The address is coded consecutively, and each storage unit has only one successor unit. When the data node *N* of the logical structure is stored in the computer, it is necessary to establish a mapping relationship between the logical node and the storage structure, that is, to establish the mapping relationship between the logical data and the physical storage.

A flowchart is a common way to describe logical processes in engineering design and software development^9–11^. A node in the flowchart may have multiple inputs or multiple outputs. It is thus inappropriate to express in a linear structure. Current flowchart logical structure is mainly represented by graphs, including adjacency lists, cross-linked lists, and adjacency matrices. However, nodes in flowcharts are connected in an orderly fashion, rather randomly, that is, the inflow and outflow of nodes have regularities. But the traditional adjacency list, cross-linked list, and adjacency matrix include undirected graphs as well as arbitrary connections of directed graphs. This causes many problems. For example, consider a graph that contains 100 nodes and many edges that connect the nodes. Suppose the nodes can be arbitrarily connected by edges, and each connection type has a unique characteristic. This can be represented by the adjacency table data structure. However, if these nodes are arranged to follow certain connection rules, like the process in a pipeline or the flow of a river, which always flows downstream, and there may be branches at the downstream, then the adjacency table data structure becomes very inefficient.

### Adjacency matrix

The adjacency matrix representation of a graph includes a node table that stores the information of each node (vertex) and an adjacency matrix that describes the relationship among the nodes. Storing a flowchart *G* with *n* nodes and *e* edges in an adjacency matrix requires a sequence table with *n* nodes and an $n \times n$ adjacency matrix, resulting in a storage capacity of $n+n \times n=n^2+n$. Thus, when a graph is represented by an adjacency matrix, the storage space is a function of the number of nodes in the graph, but not the number of edges. When it is used to store a sparse graph, there will be a large number of zero elements, and storing these zero elements will consume a lot of storage space. An example of an adjacency matrix structure is shown below

$$\begin{aligned} \left[ \begin{array}{cccc} 0 &{} 1 &{} 0 &{} 1 \\ 1 &{} 0 &{} 1 &{} 0 \\ 0 &{} 1 &{} 0 &{} 1 \\ 1 &{} 0 &{} 1 &{} 0 \\ \end{array} \right] \end{aligned}$$

where the numbers 0’s and 1’s represent the connection relationship between the two nodes: 0 means no connection and 1 otherwise.

The number of 0’s and 1’s in the adjacency matrix determines its time complexity, which is the product of the number of nodes on the rows and the number of nodes on the columns ${{\mathcal {O}}}(n^2)$.

### Adjacency list

The adjacency list is another common graph representation method. Its storage space is a function of both the number of nodes and the number of edges. An adjacency list is a chained storage structure for a graph. It consists of a sequentially stored node list and *n* singly-linked edge lists. The entries in the node table have a one-to-one mapping with the nodes in the graph, and each entry includes two fields, which are used to store the node data and the pointer to the edge table of the node, respectively. The entries in the edge table also have two fields to store the sequence number of another node associated with the edge and a pointer to the next entry in the edge table. The adjacency list structure is as follows.

**Table:** 

vertex	firsArc	$\rightarrow$	no	next

Flowcharts represented by the adjacency list data structure require a storage capacity of $n+e$. Compared with the adjacency matrix, the adjacency list significantly reduces storage space. The adjacency list time complexity is ${{\mathcal {O}}}(n+e)$, determined by the sum of the number of nodes and edges.

### Cross-linked list

The cross-linked list is similar to the adjacency list; the difference is that both the inflow and outflow relationships between nodes and edges are expressed in a table structure. This means that the adjacency list and the inverse adjacency list of the flowchart are combined to store the flowchart. The cross-linked list structure also consists of a node table and an edge table. Each entry in the node table consists of a node data field and an edge pointer field. The edge pointer field is divided into two: the first one points to the first edge to which this node flows out; the second points to the first edge that ends at this node, that is, the first edge that flows into this node. The edge table consists of a mark field, a node serial number field *i*vex and *j*vex, and a link pointer field *i*link and *j*link. The mark field is used to mark whether the edge has been processed or searched for; *i*vex and *j*vex are the two node numbers associated with the edge. The link pointer *i*link points to the next entry with the same starting point as the current edge; *j*link points to the next edge whose end-point is the same as this edge. The cross-linked list structure is as follows.

**Table:** 

vertex	firstIn	firstOut		
mark	*i*vex	*j*vex	*i*link	*j*link

The use of a cross-linked list to represent a flowchart is similar to the use of an adjacency list, with node and edge pointers as the only link relationship, and each node or edge in the node and edge lists has a separate pointer. In terms of storage capacity and time complexity, it is also similar to the adjacency list. Further graph data structures including the adjacency list and the cross-linked list structures can be found in^21–23^.

### Discussion

In the adjacency matrix representation, it is convenient to determine whether there is an edge between two nodes by the value of an element in the matrix. Problems of the adjacency matrix representation are storage space and traversal time. The adjacency list and cross-linked list require scanning the node in the edge table to determine the flow direction. They are more complex, but can reduce the storage space and time complexity to a certain extent. However, there still is a room for optimization.

AI algorithms have seen an ever increasing number of applications recently, but the required computing power and storage space are scaling up dramatically as well. Flowcharts are widely use in AI^8,17,24^. Thus the power requirement for the flowchart mandates its logical structure optimization for faster traversal speed and less storage space.

## New data structures for flowchart design

### Existing data structures for graph

When the adjacency list, cross-linked list, and adjacency matrix logical structures are used to describe a flowchart, there exist null elements for non-connected items. When the data is converted into a storage structure, these null elements will increase both storage space and unnecessary running time overhead. A brief summary of existing common structures for flowchart design is given in Table 1. Next we present three examples of the storage structures of the adjacency list, adjacency matrix, and cross-linked list for flowcharts.

**Table 1:** Summary of common structures for flowchart design.

Methods	Features	Advantage	Disadvantage
Adjacency matrix	It consists of a node-table, which stores node information, and a matrix, which stores the connection relation between nodes. Its memory size $(n^2)$ depends only on the number of nodes (*n*)	The adjacency relationship between all nodes is very clear	The storage space occupied is large
Adjacency list	It consists of a node table and n edge-tables. Each node has an edge-Table. The node table stores information and pointers that point to the edge-table for each node. The edge table of each node stores the serial number of another node (which is associated with this node) and a pointer, which points to the next edge table	The storage space occupied is smaller relative to the adjacency matrix	The adjacency relationship among all nodes is much more difficult to identify than the adjacency matrix
Cross-linked list	Based on the adjacency list, it sets two pointers in the node table. The first pointer points to the first edge starting from this node, and the second pointer points to the first edge ending from this node	It occupies the same storage space as the adjacency list	The adjacency relationship among all nodes is similar to adjacency list

#### Adjacency list example

**Figure 1:**
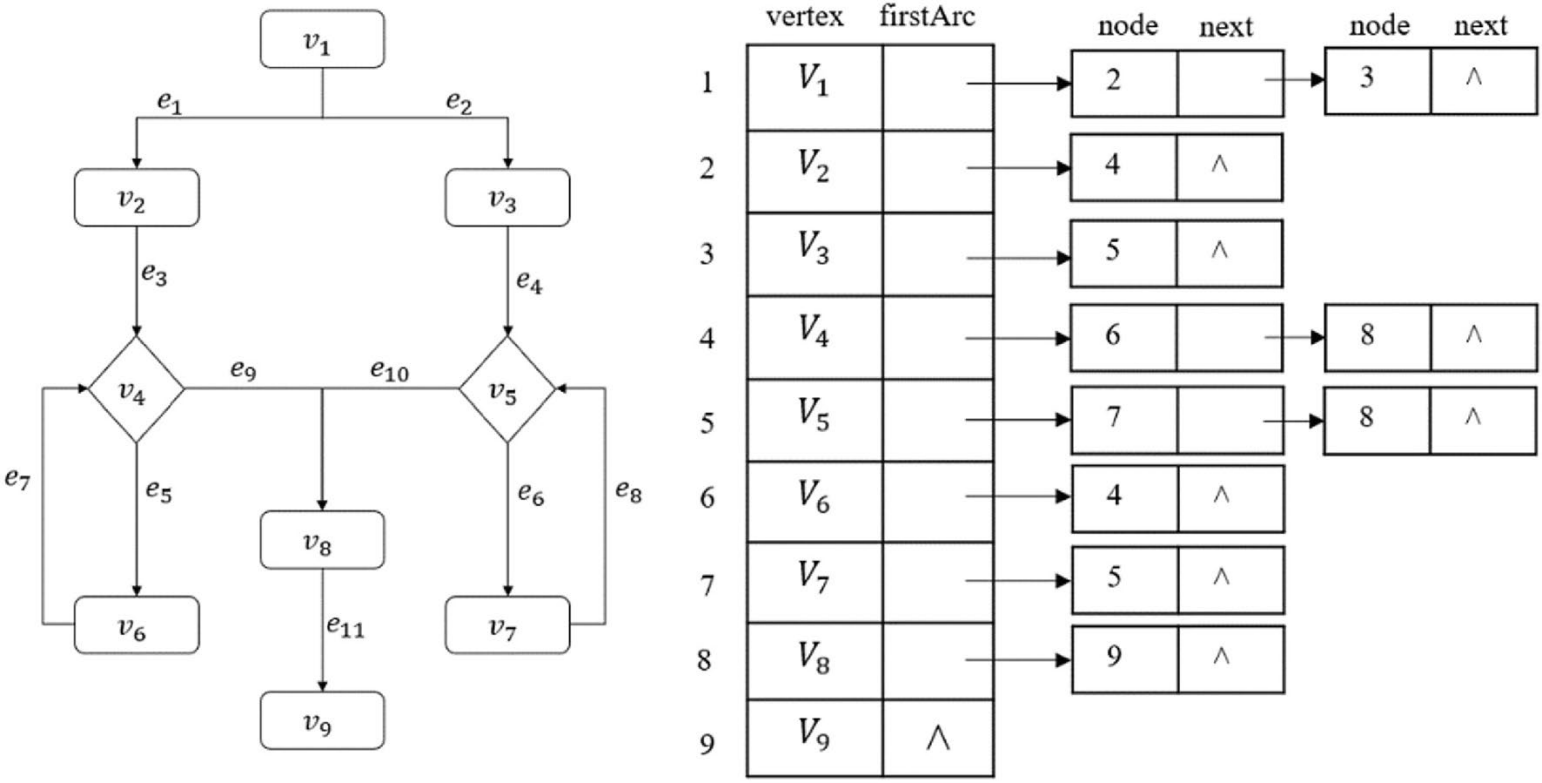
A flowchart represented by the adjacency list structure (flowchart on the left; adjacency list on the right).

An example of the adjacency list structure is shown in Fig. 1.

As shown on the left of Fig. 1, the directional connecting lines represent the logical relationship between the nodes, that is, the adjacency relationship. Shown on the right of Fig. 1 is the adjacency table corresponding to the flowchart on the left: $V_1$ to $V_6$ correspond to the nodes represented by subscripts $1-6$, respectively. The two columns, ‘vertex’ and ‘firstArc’, represent, respectively, the node data and the pointer to the first edge. Each row of ‘vertex’ and ‘firstArc’ forms a node entry, each occupying one storage unit. The first ‘node’ and ‘next’ columns on the right of Fig. 1 represent, respectively, the next node to which the first edge-pointer points and the next edge pointer. Each entry of these two columns constitutes an edge entry; the second ‘node’ and ‘next’ columns respectively represent the next node to which the second edge-pointer points and the next edge pointer. In the same way, each row with multiple fields in the two columns of ‘node‘ and ‘next‘ forms an edge entry which occupies one storage unit. The symbol $^\wedge$ means that it does not exist, i.e., a null value. In Fig. 1, there are a total of 9 nodes and 11 edges, occupying a storage space of $C_L=9+11=20$.

#### Adjacency matrix example

**Figure 2:**
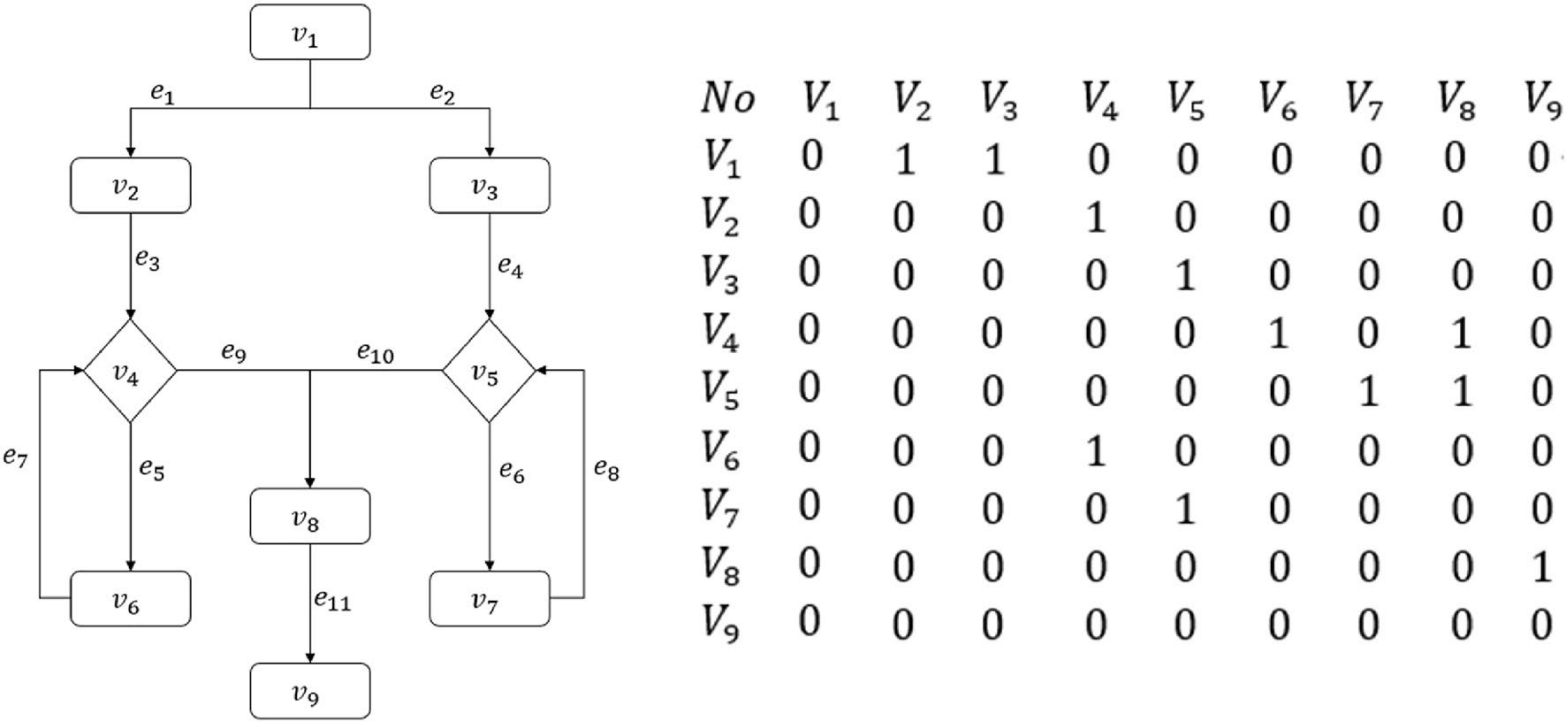
A flowchart represented by the adjacency matrix (flowchart on the left; adjacency matrix on the right).

Figure 2 shows an adjacency matrix representation of a flowchart. Entries of the first row and first column of the matrix are node labels (i.e., $V_1, \ldots , V_9$). Other entries represent the edges between nodes. For example, the (2, 3)-entry is the edge from node 1 to node 2, denoted as $\overrightarrow{V_1 V_2}$. The value ‘1’ of each entry means that there is an edge; ‘0’ means no edge. Whether there is an edge or not is determined by the direction of flow. In this example, there are $n=9$ nodes, occupying 9 storage units, and a total of $E=n^2$ 1’s and 0’s. Each 1 or 0 occupies one storage unit. Thus the total number of storage units is $C_M=E^2+n=9^2+9=90$.

#### Cross-linked list example

**Figure 3:**
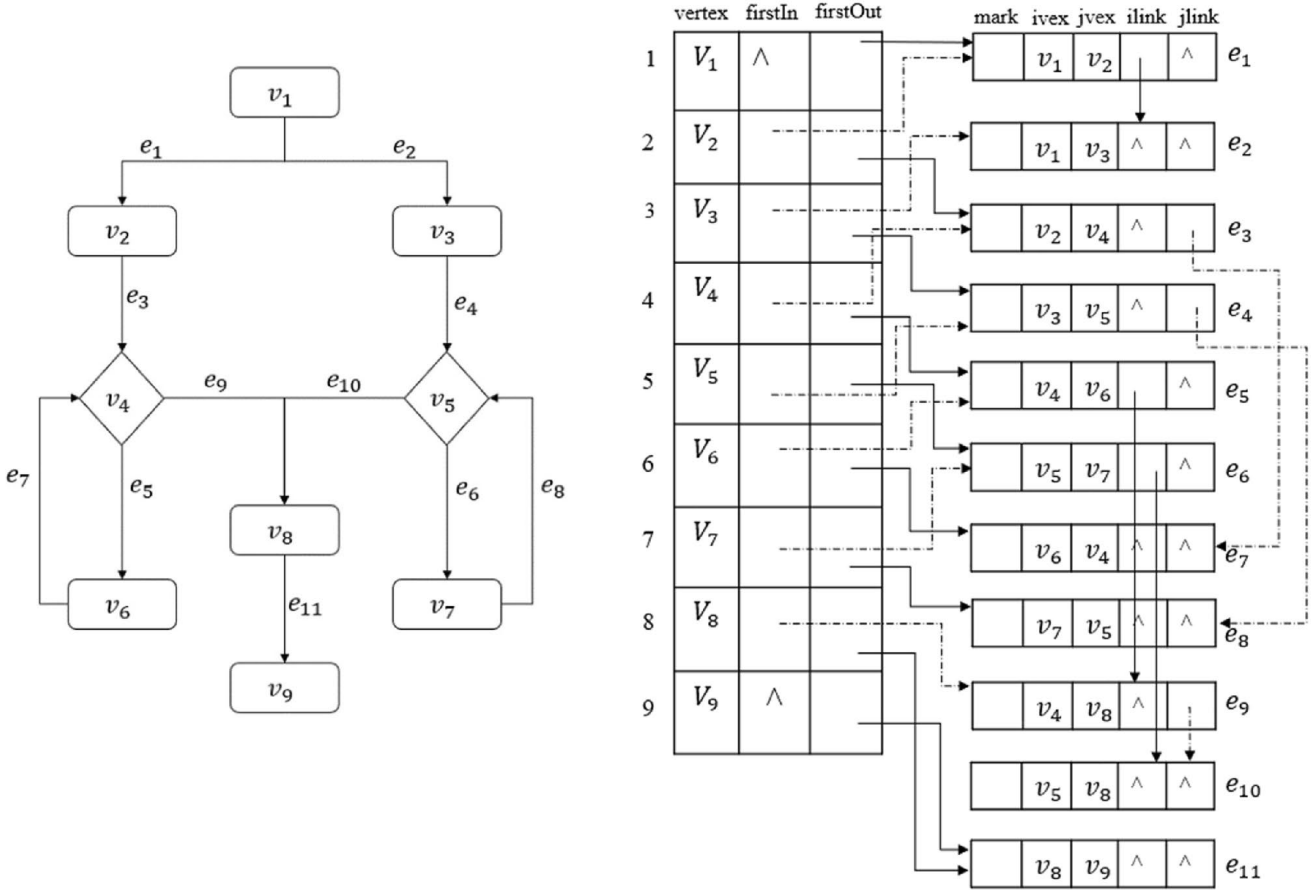
A flowchart represented by the cross-linked list (flowchart on the left; cross-linked list on the right).

Figure 3 is the cross-linked list representation of a flowchart.

The storage space used by the cross-linked list is the same as that of the adjacency list, but the representation is more complex. An advantage of the cross-linked list representation is that it can clearly express the edges flowing into and out of the same node in the same graph. When there exist conditions and loops in the flowchart, one could specify the loop relationship and the number of loops in the ‘mark’ field. The node with conditions can be determined through the connection relationship between the node table and the entries in the edge table. The cross-linked list is more efficient than the adjacency list in storing flowcharts with loops and conditions.

With either the adjacency matrix, or adjacency list, or cross-linked list, it is difficult to describe loops between nodes concisely and clearly. Additionally, complex flowcharts could have sub-flowcharts nested in a node, and the sub-nodes in the sub-flowcharts could further have sub-flowcharts nested in them, and so on. With such a layer-by-layer nesting relationship, the adjacency matrix, adjacency list, and cross-linked list structures will become extremely complex, and the storage space and time complexity will become excessive.

### Hierarchical data structures for flowcharts

**Figure 4:**
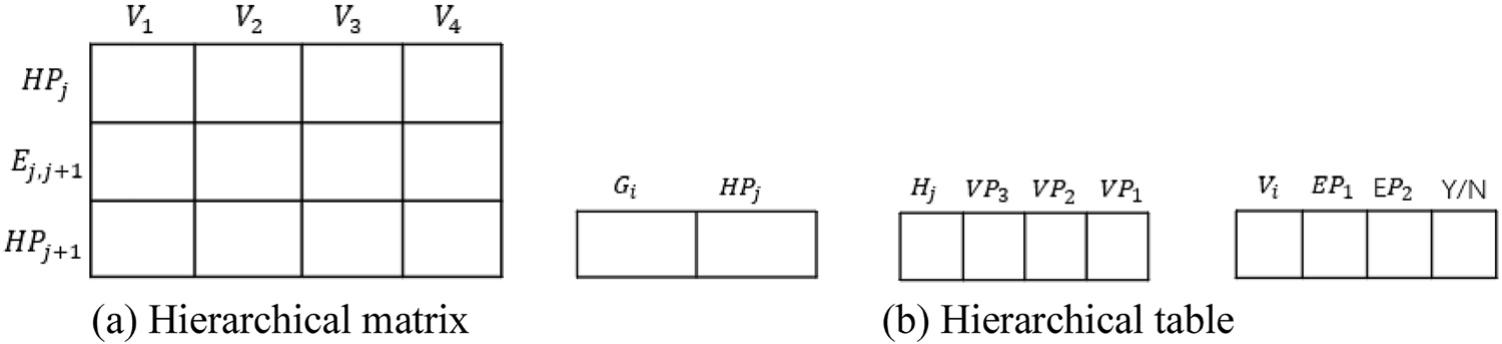
Hierarchical data structures for flowcharts.

To resolve the above problems, we design two new data structures for flowcharts that can effectively and efficiently express both the sequential and nesting relationships among the nodes: a hierarchical matrix data structure and a hierarchical table data structure as illustrated in Fig. 4.

#### Hierarchical matrix data structure

In the hierarchical matrix data structure, the row $HP_j$ is the hierarchy, and the row $E_{j,j+1}$ is the connection of each node from the $HP_i$ layer to the $HP_{j+}$ layer, i.e., the edge.

**Figure 5:**
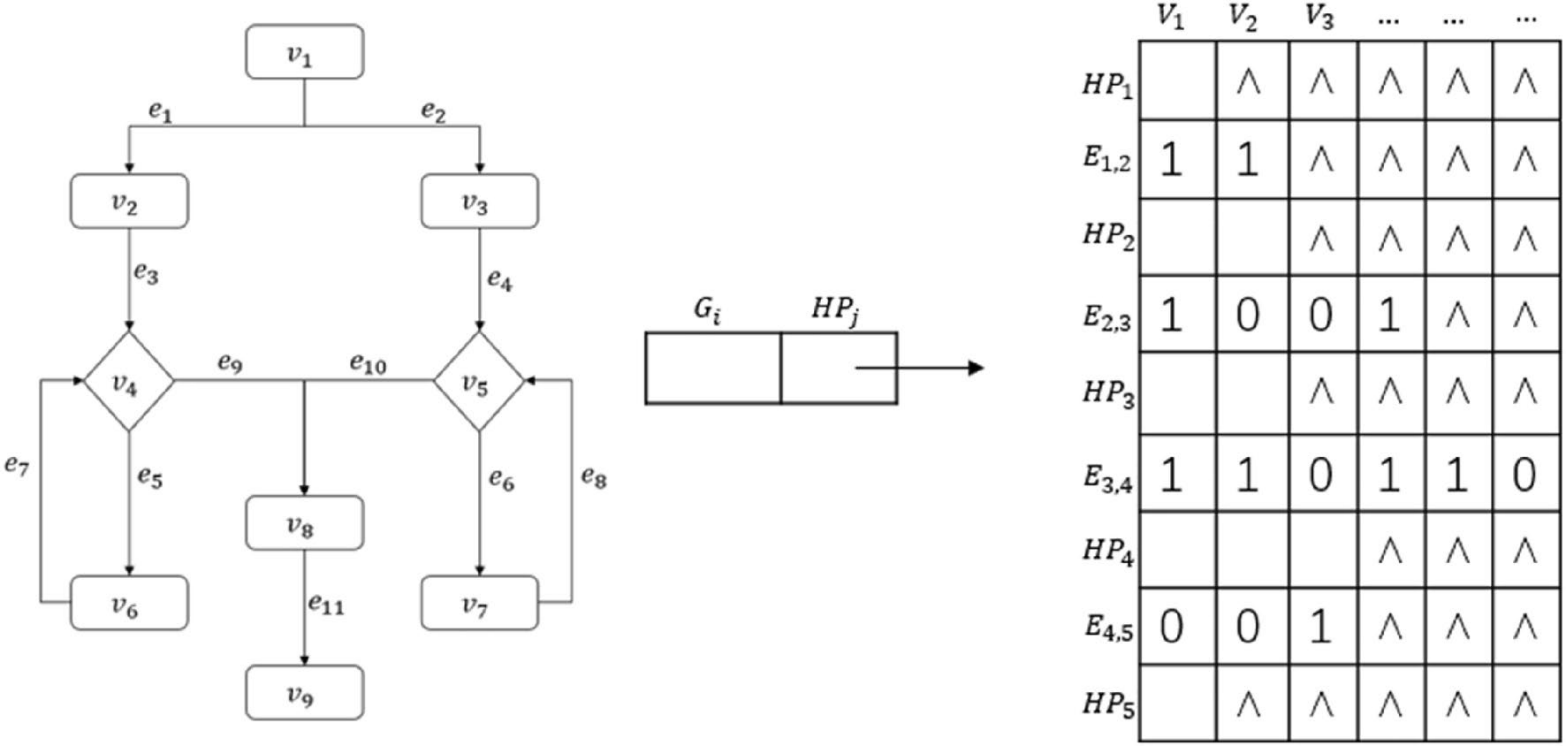
Hierarchical matrix structure.

Figure 5 shows an example of the hierarchical matrix table, where ‘1’ indicates that the nodes between the upper and lower layers have a connection while ‘0’ means none. The number of connections is the product of the numbers of nodes in the two layers. In the example, $HP_1$ layer has one node, and $HP_2$ layer has two nodes. Thus there are $1\times 2=2$ connections in the $E_{1,2}$ row. Similarly, there are 2 nodes in $HP_4$ layer and 3 nodes in $HP_5$ layer; thus there are $2 \times 3=6$ connections in $E_{3,4}$ rows. The maximum number of 6 connections used is the number of columns of the matrix, the symbol $^\wedge$ means a null value, the $E_{j, j+1}$-th row has a ‘1’ or ‘0’ connection relationship from left to right is: $HP_j$ layer has 2 nodes and $HP_{j+1}$ layer (the $(j+1)$-th layer) has 3 nodes, the first node in the $HP_j$ layer is connected to the first, the second, and the third node in the $HP_{j+1}$ layer. The second node in the $HP_j$ layer is connected to the first, the second, and the third node in the $HP_{j+1}$ layer.

**Figure 6:**
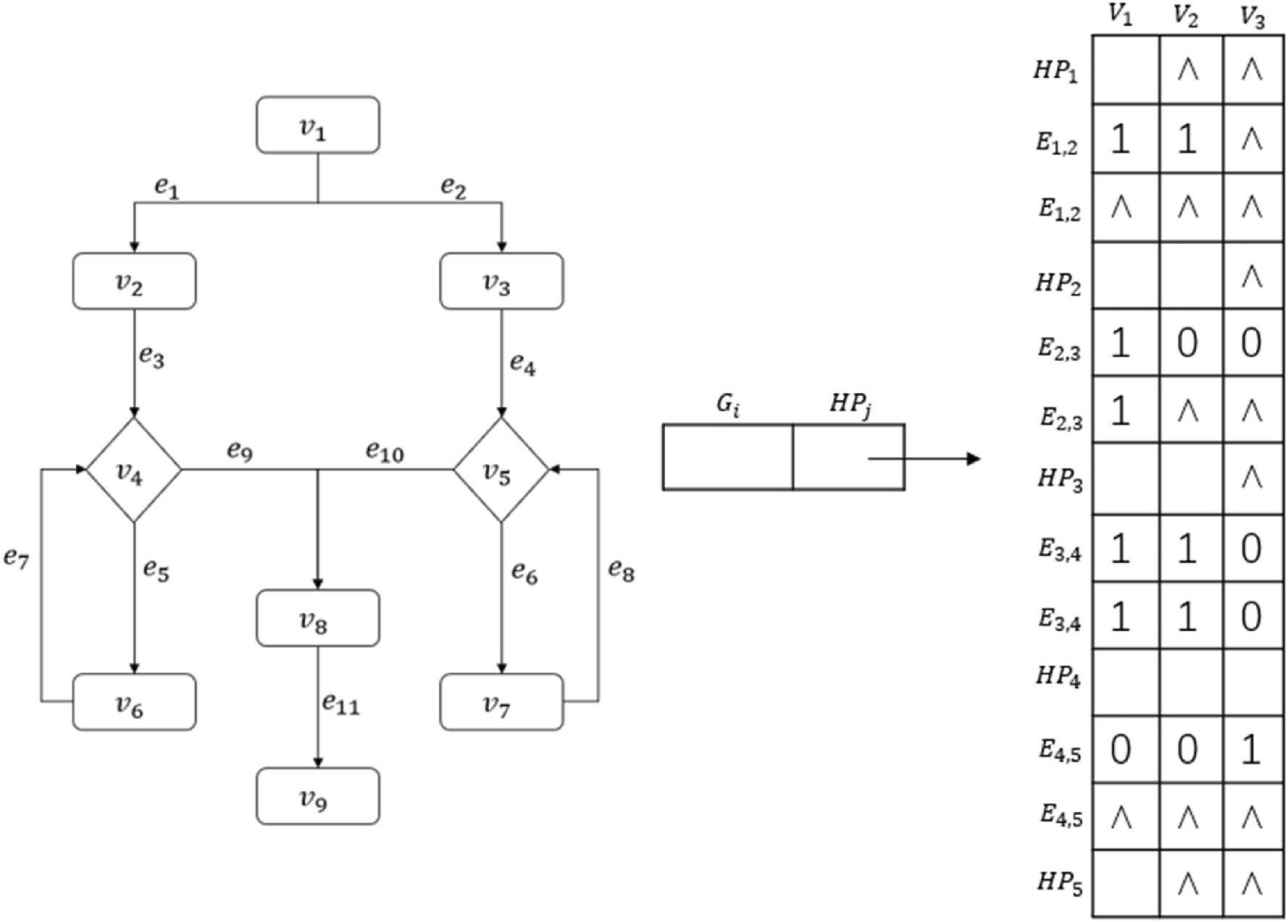
A transformed hierarchical matrix structure.

In Fig. 5, there are 9 rows and 6 columns of cells (memory cells), totaling 54. In order to more clearly represent this data structure with a matrix and further optimize the storage space, each $E_{j,j+1}$ row is decomposed into multiple rows. The number of rows is the maximum number of node connections in $E_{j,j+1}$ divided by the maximum number of nodes in each layer. In the example in Fig. 5, the maximum number of connections is 6, and the maximum number of nodes in each layer is 3; thus the number of rows to be decomposed into is $6 \div 3=2$, as shown in Fig. 6, a transformed hierarchical matrix structure. After this optimization, the matrix has 13 rows and 3 columns of cells, totaling $13 \times 3=39$ storage cells.

**Figure 7:**
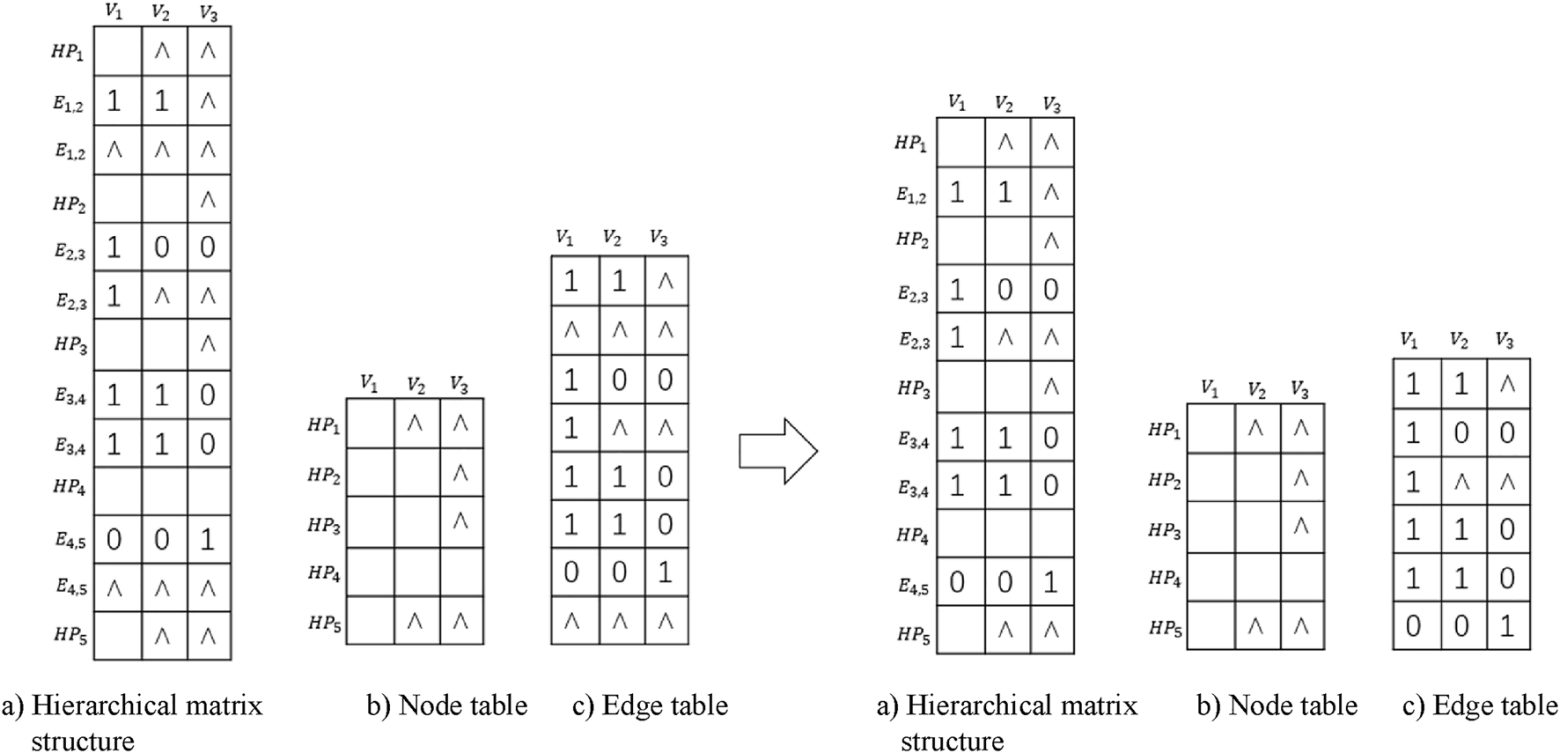
Decomposed form of the hierarchical matrix structure.

If the connection relationship between the upper and lower layers have several null values, then the structure should be further optimized, as shown in Fig. 7.

**Figure 8:**
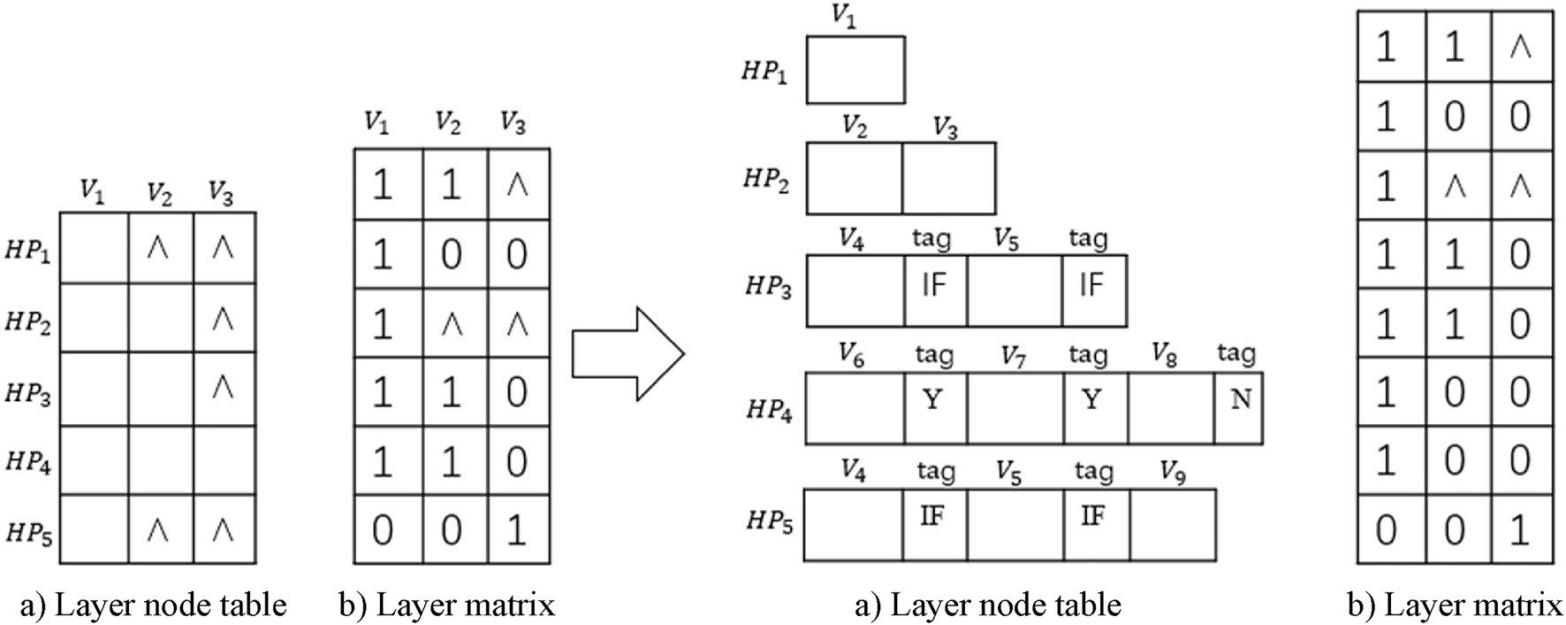
Hierarchical matrix structure with loops.

Figure 8 shows a hierarchical matrix structure with nodes that have loops. Here decision parameter *Y* and *N* are included, where *Y* denotes “conditional Yes” and *N* means “does not meet the condition No”. If the decision label is *Y*, then the loop continues; otherwise the loop stops and starts the next node.

#### Hierarchical table data structure

**Figure 9:**
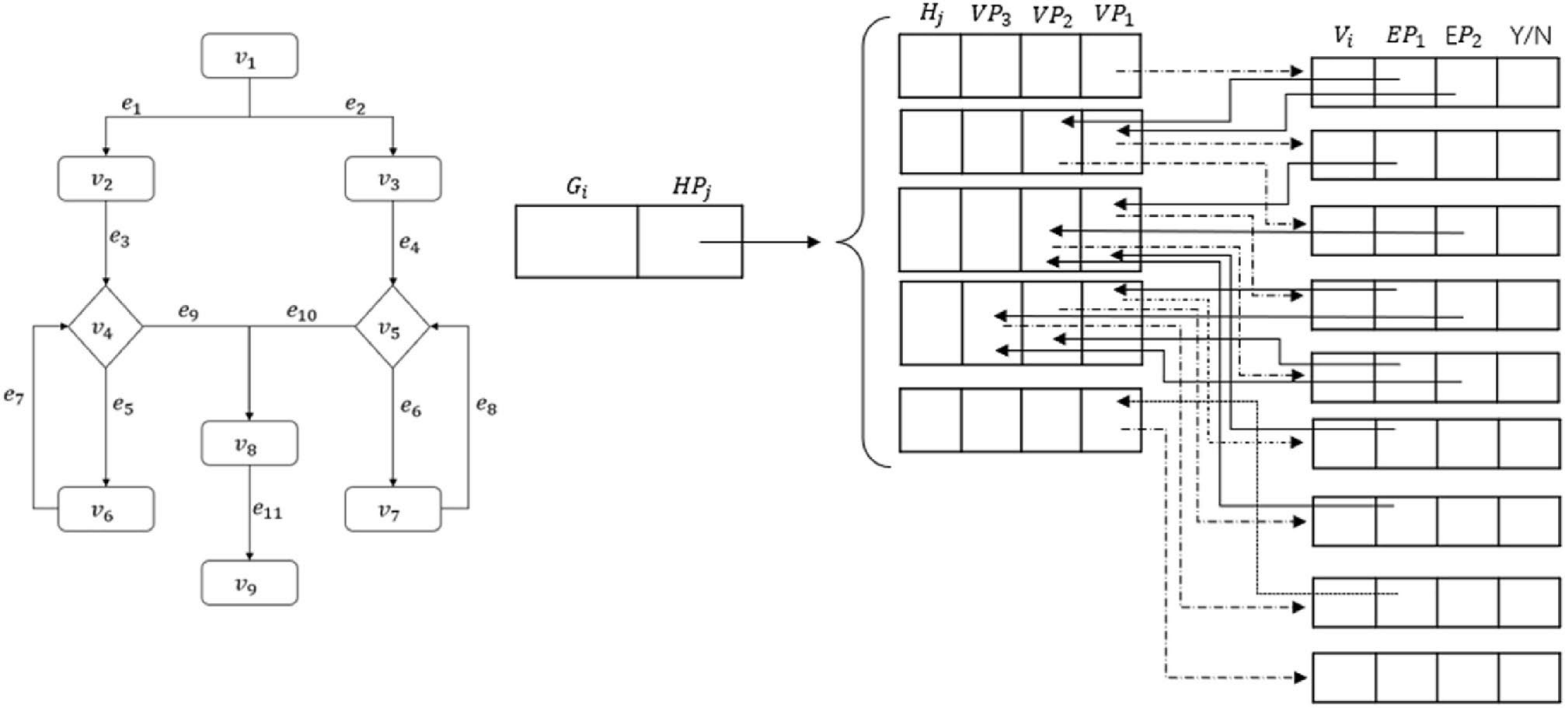
Hierarchical table structure.

Figure 9 shows a hierarchical table structure for flowcharts.

**Figure 10:**
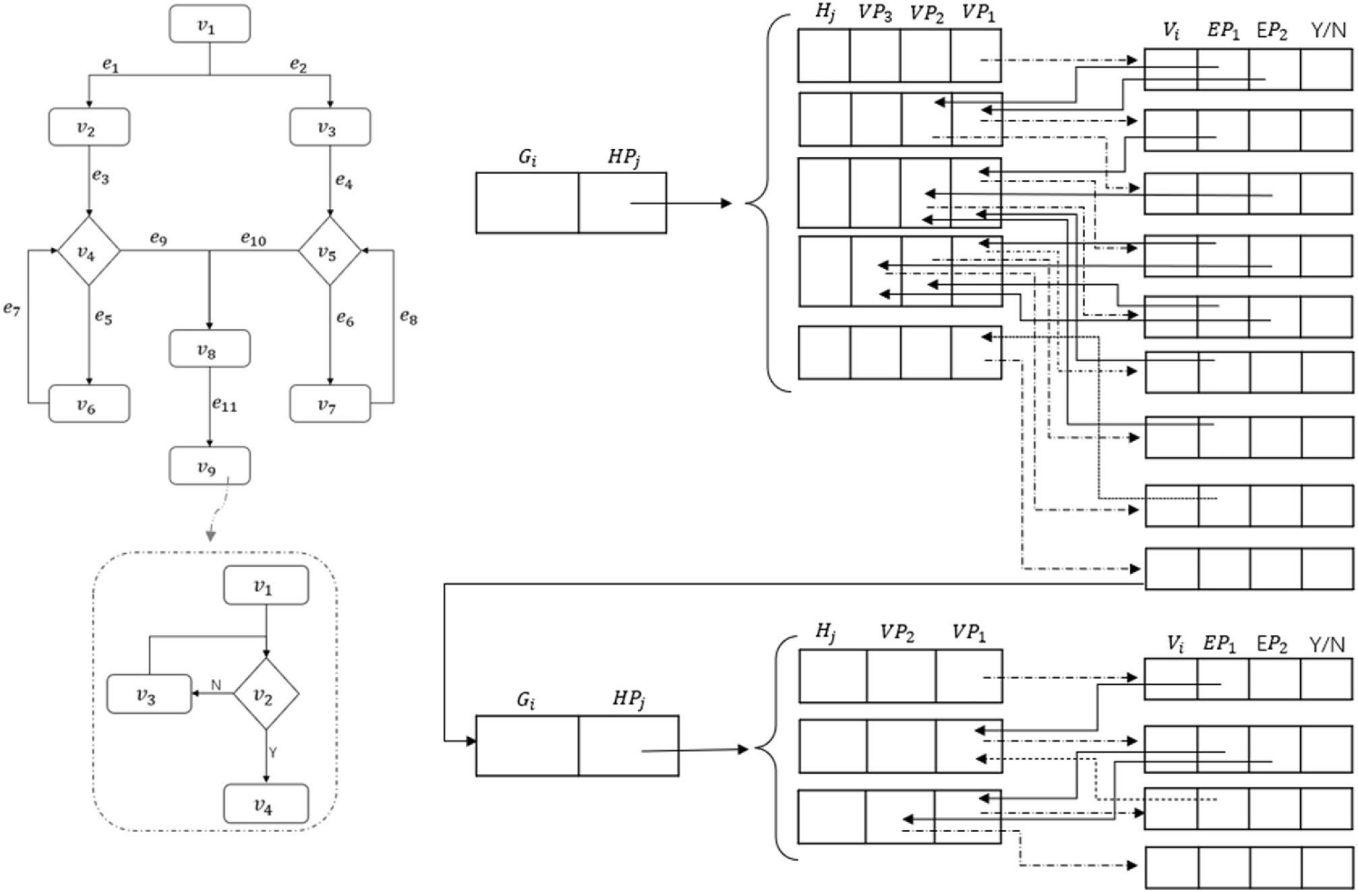
Hierarchical structure of flowcharts with node groups.

Figure 10 is an example of a flowchart hierarchy that includes node groups. It is assumed that node 9 is a node group, that is, a sub-flowchart is nested in node 9. The bottom-left is the nested sub-flowchart of node 9.

### Hierarchical traversal processes

#### Analysis

**Figure 11:**
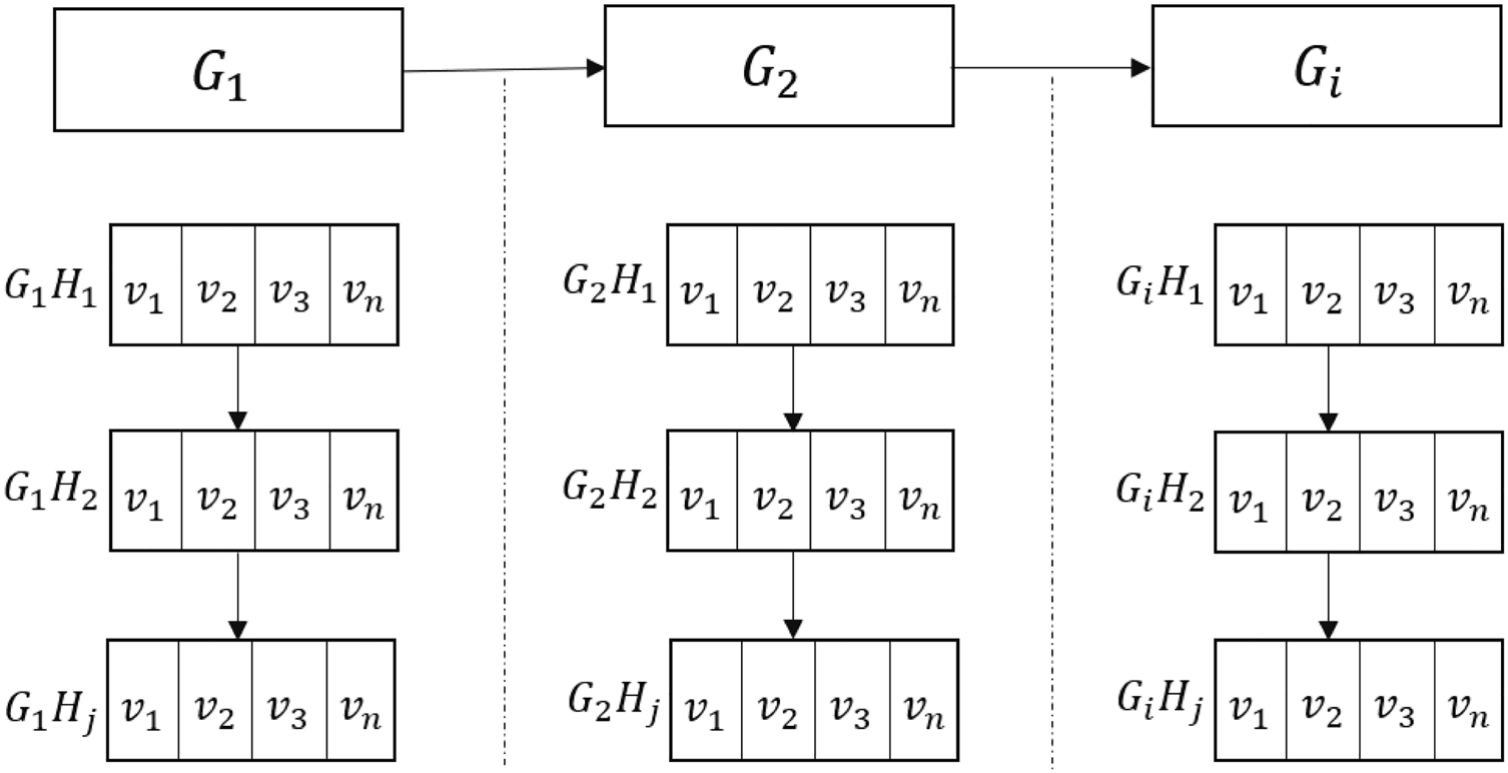
Hierarchical traversal method.

The hierarchical traversal method is shown in Fig. 11, where $G_1, G_2, \ldots , G_N$ are level-node groups, $G_2$ is nested in $G_1$, $G_{i+1}$ is nested in $G_i$. $G_2$ and $G_i$ each can have multiple level-node groups. For example, $G_2$ can have $G_{21}$ and $G_{22}$. Each level-node group is composed of multiple layer-node groups; $H_1, H_2, \ldots , H_N$ are the layer-level groups of each level-node group; $v_1, v_2, \ldots , v_N$ are the nodes of each layer-node group.

The hierarchical method traverses from $G_1$ to $G_2$, then to $G_i$; traversal of $G_1$ goes from $H_1$ to $H_2$, and then to $H_j$. After all layer-node group traversals are completed, the process jumps to the layer-node group of the nested level-node group; $G_2$ and $G_i$ follow the same process. Traversal of $H_1$ goes from $v_1, v_2, \ldots , v_n$ according to the node order. When all nodes in $G_1$ are traversed, the process finally jumps to the node of the nested level-node group and starts traversing $G_2$. If there are multiple nested level-node group nodes, then traverse them one by one, for example, first $G_{21}$ and then $G_{22}$.

#### Comparison of breadth-first, depth-first, and hierarchical traversal methods

**Figure 12:**
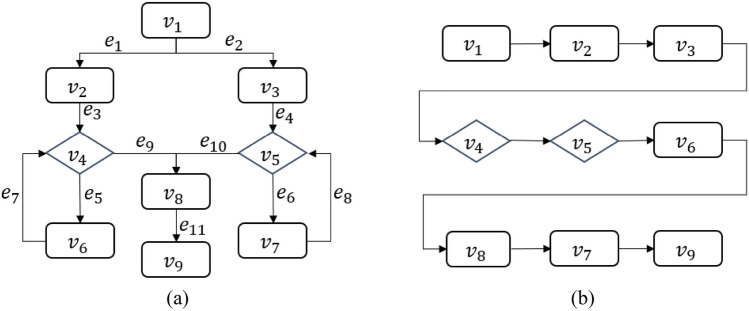
(**a**) Flowchart example; (**b**) Node sequence of breadth first traversal.

Here we use specific examples to compare the various traversal methods. An example of the vertex sequence of breadth-first traversal is shown in Fig. 12. First visit vertex $v_1$, then visit, in turn, all unvisited vertices $v_2$ and $v_3$ adjacent to $v_1$, and then visit all unvisited vertices adjacent to $v_2$ and $v_3$ and so on. If there are still unvisited vertices, then choose one of them as the starting vertex and repeat the process until all the vertices in the graph have been visited.

**Figure 13:**
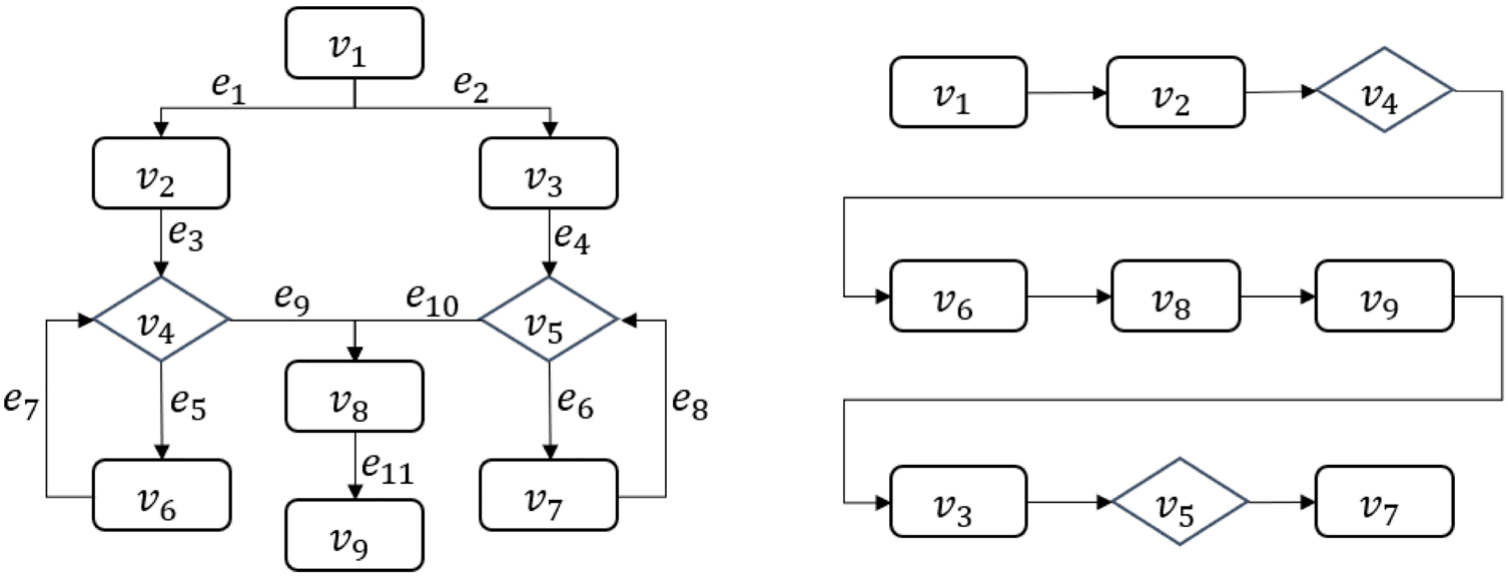
Example of node sequence of depth-first traversal for flowchart.

An example of the vertex sequence of depth-first traversal is shown in Fig. 13. First visit the vertex $v_1,$ then select a vertex $v_2$ that is adjacent to $v_1$ and has not been visited, and then start from $v_2$ and continue to traverse the vertex $v_4$ that is adjacent to $v_2$ and has not been visited, and so on. When the traversal gets to vertex $v_6$ whose adjacent vertices all have been visited, return to the last vertex $v_1$ in the visited vertex sequence that has an unvisited adjacent vertex $v_3$, and then start from $v_1$ and traverse $v_3$. This process is repeated until all vertices in the graph have been visited.

For a graph with *n* vertices (nodes) and *e* edges, using the adjacency list storage structure, and adopting depth-first or breadth-first traversal, all vertices in the vertex table need to be visited once, and the entries in the edge table is scanned once. Therefore, its time complexity is ${{\mathcal {O}}}(n+e)$. If the adjacency matrix is used as the storage structure, the the time required to find all the edges of each vertex is ${{\mathcal {O}}}(n)$, and the time required to traverse all the vertices in the matrix graph as shown in Fig. 5 is ${{\mathcal {O}}}(n) \times {{\mathcal {O}}}(n) = {{\mathcal {O}}}(n^2)$.

**Figure 14:**
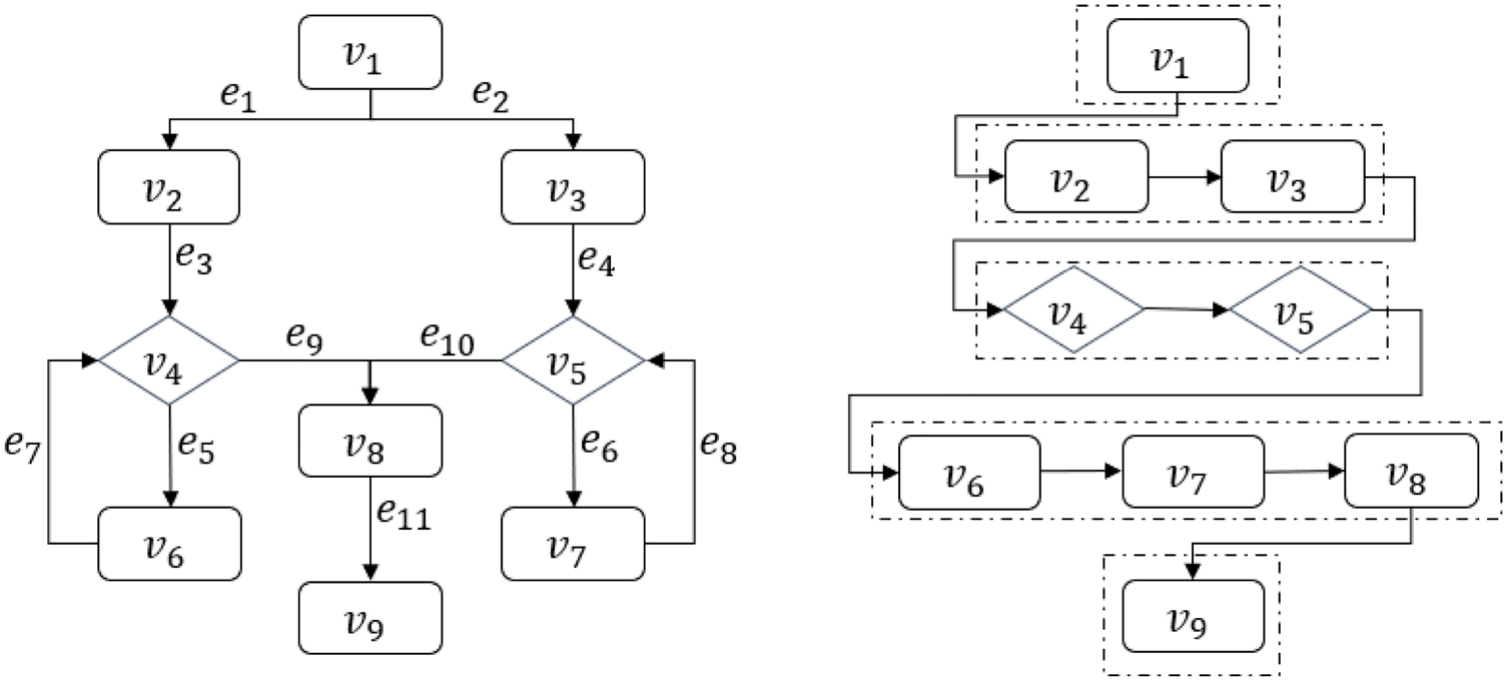
Hierarchical node traversal sequence (each dashed box represents a layer).

An example of the vertex sequence of hierarchical traversal is shown in Fig. 14. This flowchart has only one level $G_1$. Traversal starts from the first layer of $G_1$, and the vertex in the first layer is $v_1$. After $v_1$, it traverses the second layer $(v_2, v_3)$, then the third layer $(v_4, v_5)$, then continues to traverse the fourth layer $(v_6, v_7, v_8)$, and finally traverse the fifth layer $(v_9)$, until all layers are traversed. Now the traversal process looks for nodes in the layer-node group that have nested level-node groups. Since in this flowchart example no layer-node groups have nested level-node group.

## Analysis of the proposed data structures

The space complexity is defined as the storage space, and the time complexity is defined as the time efficiency when traversing through the flowchart. There are two commonly used graph traversal methods: depth-first traversal and breadth-first traversal^25,26^. Depth-first traversal is similar to the root traversal of a tree. It starts from the starting node, then selects an adjacent and unvisited node, and then recursively searches for adjacent nodes from this node. For cases when all nodes have been visited, it selects an unvisited node as the starting node to continue traversing until all nodes are traversed. Breadth-first traversal starts from the starting node, then visits all unvisited nodes adjacent to this node one by one, and then visits the unvisited adjacent nodes of the next node, until all reachable nodes have been visited. If there exist unvisited nodes, then another unvisited node is selected as the starting point to continue traversing. The above processes are repeated until all nodes in the graph are visited.

For the hierarchical table and hierarchical matrix methods, the traversal methods can be either breadth-first or depth-first. In order to further optimize the time complexity, and to deal with cases with node groups, this paper also designs a level- and layer-traversal method, here named as “hierarchical traversal”. The details are as follows.

First determine the node of a level, the top-level is treated as a node, that is, a “level-node group”. Then determine the node of the layer, and treat all nodes in each layer as a node, that is, a “layer-node group”. The layers are ordered and numbered, and further determine the connection relationship between the nodes in the previous layer and the nodes in the adjacent next layer. The highest time complexity of the nodes traversing the connections between two adjacent layers is ${{\mathcal {O}}}(m \times n)$, where *m* is the number of nodes in the upper layer, *n* is the number nodes in the lower layer of the adjacent nodes.

Here are the traversal criteria to optimize the time complexity of traversing through the nodes in two adjacent layers: the nodes in each layer are labelled numerically (in increasing order). When there is only one node in one of the two adjacent layers, the connection is one-to-many by default; when there are multiple and equal number of nodes in both layers, the connection between nodes of the two layers would be first-to-first, second-to-second, and so on. Then we must judge whether the number of edges conforms to a pre-determined number; if not, then start from the node with the largest label (a number) in the upper layer to determine the connection relationship between it and the node with the second largest sequence number in the lower layer, and check the edge conditions. This process continues and traversing stops until the edge condition is met. If none of the cases meets the edge condition, then start with the second largest sequence number in the upper layer and go through the same process. An identifier is added for condition-checking nodes. Also, for condition-checking nodes (a class) that flow out of the upper-layer but flow into the same class in the upper layer, a backflow identifier should be added for them. Nodes with a backflow identifier do not participate in the process of checking whether or not to traverse to the next layer.

### Hierarchical table data structure analysis

Take the flowchart in Fig. 5 as an example. With the hierarchical traversal method, the sequence numbers of the nodes are: $H_1$: $v_1$, $H_2$: $v_2, v_3$, $H_3$: $v_4, v_5$, $H_4$: $v_6, v_7, v_8$, $H_5$: $v_9$. The layers are numbered in order; the first layer has only one node, and the second layer has two nodes. Based on the design rules, $v_1$ flows to $v_2$ and $v_1$ also flows to $v_3$ by default. The third layer has two nodes. By default the first node of the second layer $v_2$ flows to $v_4$ of the third layer, and the second node of the second layer $v_3$ flows to $v_5$ of the third layer; $v_4$ and $v_5$ of the third layer are condition-checking nodes. The fourth layer has three nodes. By default the first node of the third layer flows to the first node of the fourth layer, and the second node of the third layer flows to the second node of the fourth layer. Now the number of edges is two fewer than necessary. Thus check whether the second node of the third layer actually flows to the second node of the forth later. If true, and the number of traversed edges has reached three, then continue traversing the first node of the third layer and the third node of the fourth layer. At this point, the number of edges traversed is four, which meets the condition. Hence the traversal of the fourth and fifth layers begins. Since $v_6$ and $v_7$ of the fourth layer have backflow identifiers, they do not participate in the traversal to determine if whether they flow into the next layer or not, leaving only $v_8$ and $v_9$ of the fifth layer. By default $v_8$ flows into $v_9$, and $v_9$ is the bottom layer, thus ending the traversal of the flowchart.

The traversal time complexity of the flowchart in Fig. 5 is mainly determined by the third and fourth layers. The two added traversal edges *n* and the number nodes participated in the traversal *m* are indicators of the time complexity between the two layers. The time complexity of the third layer and the fourth layer is ${{\mathcal {O}}}(n+m)={{\mathcal {O}}}(5)$, and the total traversal time complexity of the five layers is roughly estimated as

1 $$\begin{aligned} {{\mathcal {O}}}(T_s)={{\mathcal {O}}}(C_H+{{\mathcal {O}}}(n+m))={{\mathcal {O}}}(5+5)={{\mathcal {O}}}(10), \end{aligned}$$

where $C_H$ is the number of layers. If the adjacency list structure is used to store the flowchart in Fig. 5, all nodes in the node table need to be visited once, and the entries in the edge table need to be scanned once. Thus the traversal time complexity is

2 $$\begin{aligned} {{\mathcal {O}}}(e+N)={{\mathcal {O}}}(11+9)={{\mathcal {O}}}(20), \end{aligned}$$

where *N* is the total number of nodes and *e* is the total number of edges.

These results show that, based on the flowchart in Fig. 5 as an experimental example, the ratio of time complexities of the hierarchical table and the adjacency table is $R_{ta} = \frac{{{\mathcal {O}}}(C_H + {{\mathcal {O}}}(n=m))}{e+N} = \frac{10}{20} = 0.5$. The increase in time efficiency is $A_{da}=\left( 1-R_{ta}\right) \times 100\% = \left( 1-0.5\right) \times 100\%=50\%$. The above analysis shows that the time complexity of the hierarchical traversal is significantly lower than that of the traditional methods.

If the hierarchical table structure is used, the space complexity (i.e., storage space) of the example shown in Fig. 5 is $TS_c=n+e=11+9=20$.

### Hierarchical matrix data structure analysis

If we adopt the adjacency matrix storage structure for the example in Fig. 5, all edges of each node must be searched, and all cells in the adjacency matrix (i.e., the nodes) must be traversed. The traversal time complexity is

3 $$\begin{aligned} {{\mathcal {O}}}(N^2)={{\mathcal {O}}}(9^2)={{\mathcal {O}}}(81). \end{aligned}$$

This is very high. If the hierarchical traversal method is adopted, traversing the flowchart Fig. 5 is equivalent to traversing the hierarchical matrix table shown in Fig. 8. The product of the numbers of rows and columns of the table is traversal time complexity:

4 $$\begin{aligned} {{\mathcal {O}}}(T_m)={{\mathcal {O}}}(U_r \times U_c)={{\mathcal {O}}}(8\times 3)={{\mathcal {O}}}(24). \end{aligned}$$

This is significantly lower than the complexity in (3). Again, based on the flowchart in Fig. 5 as an example, the ratio of the time complexities of the hierarchical matrix and the adjacency matrix is $R_{tb} = \frac{{{\mathcal {O}}}(U_r \times U_c)}{N_2} = \frac{24}{81} \approx 0.3$. The increase in time efficiency is $A_{db}=\left( 1-R_{tb} \right) \times 100\% =\left( 1 -0.3 \right) \times 100\%=70\%$.

The space complexity with the hierarchical matrix being used for the flowchart shown in Fig. 5 is

5 $$\begin{aligned} MS_c&= U_r \times U_c +(U_r+N) \nonumber \\&= 8 \times 3+(5+9)=24+14=38 \end{aligned}$$

where $U_r$ is the number of layers, $U_c$ is the number of nodes in each layer, and *N* is the total number of nodes.

### Discussion

Experimental data via examples show that the hierarchical table data structure reduces the traversal time by 50% compared with the existing adjacency list while their storage spaces are similar. In specific applications the improvement depends on the characteristics of flowcharts. The above experimental data reflect well commonly used flowcharts. The specific sets of experimental data also show that the hierarchical matrix data structure reduces the traversal time by nearly 70% and saves the storage space by about 50% compared with the existing adjacency matrix, as shown in Sections “Hierarchical table data structure analysis” and “Hierarchical matrix data structure analysis”. In summary, both the hierarchical table structure and hierarchical matrix data structure can greatly improve the traversal efficiency without increasing the storage space.

## Conclusion

As AI applications continue to develop at an accelerated pace, graphical processing consumes an ever-increasing amount of power. Optimizing the storage space and traversal time of flowcharts will help mitigate the problem of insufficient computing and engineering needs. Compared with traditional adjacency lists, cross-linked lists, and adjacency matrices, the hierarchical data structures proposed in this paper can efficiently solve the multi-level nesting problem of flowcharts. They also have obvious advantages in terms of time and space complexities over existing methods, as shown by many examples presented.

Our further work will focus on exploring applications of flowcharts based on the hierarchical data structures in low-code engineering. For example, in realizing full autonomy of a large number of collaborative engineering vehicles (e.g., loaders, excavators, trucks) based flowcharts in the mine. The goal is to drive innovations in the field of low-code applications via new technologies at the “micro-technologies” level, thereby drastically improving efficiency.

## Data Availability

The datasets used and/or analyzed during the current study are available from the corresponding author on reasonable request.
